# Architectures and complex functions of tandem riboswitches

**DOI:** 10.1080/15476286.2022.2119017

**Published:** 2022-09-11

**Authors:** Madeline E. Sherlock, Gadareth Higgs, Diane Yu, Danielle L. Widner, Neil A. White, Narasimhan Sudarsan, Harini Sadeeshkumar, Kevin R. Perkins, Gayan Mirihana Arachchilage, Sarah N. Malkowski, Christopher G. King, Kimberly A. Harris, Glenn Gaffield, Ruben M. Atilho, Ronald R. Breaker

**Affiliations:** a Department of Molecular Biophysics and Biochemistry, Yale University, New Haven, CT, USA; b Department of Biochemistry and Molecular Genetics, University of Colorado, Anschutz Medical Campus, Research-1S, Aurora, CO, USA; c Department of Molecular, Cellular and Developmental Biology, Yale University, New Haven, CT, USA; d Howard Hughes Medical Institute, Yale University, New Haven, CT, USA; e PTC Therapeutics, Inc, South Plainfield, NJ, USA; f Department of Chemistry, Yale University, New Haven, CT, USA

**Keywords:** Aptamer, noncoding RNA, gene regulation, logic gate, transcription control, translation control

## Abstract

Riboswitch architectures that involve the binding of a single ligand to a single RNA aptamer domain result in ordinary dose-response curves that require approximately a 100-fold change in ligand concentration to cover nearly the full dynamic range for gene regulation. However, by using multiple riboswitches or aptamer domains in tandem, these ligand-sensing structures can produce additional, complex gene control outcomes. In the current study, we have computationally searched for tandem riboswitch architectures in bacteria to provide a more complete understanding of the diverse biological and biochemical functions of gene control elements that are made exclusively of RNA. Numerous different arrangements of tandem homologous riboswitch architectures are exploited by bacteria to create more ‘digital’ gene control devices, which operate over a narrower ligand concentration range. Also, two heterologous riboswitch aptamers are sometimes employed to create two-input Boolean logic gates with various types of genetic outputs. These findings illustrate the sophisticated genetic decisions that can be made by using molecular sensors and switches based only on RNA.

## Introduction

Since the initial reports describing the biochemical and genetic functions of metabolite-binding riboswitches [1–4], more than 50 classes have been experimentally validated [5–7]. Members of each riboswitch class are usually defined by the characteristic conserved nucleotide sequences and structural features used to form the ligand-binding aptamer structure [6,8]. In some instances, riboswitch RNAs share sequence and structural characteristics but have diversified through evolution to sense different ligands. In these latter cases, the RNAs are classified by the identity of the ligand they selectively bind (e.g. see [9–13]). Based on the number and diversity of known riboswitch classes, it has been proposed that many thousands of additional classes remain to be discovered among the various lineages of bacteria [6,14,15]. If true, these riboswitches could provide scientists and bioengineers with a robust supply of RNA devices to both study and exploit.

Already, by exploring the genetic and biochemical functions of the known riboswitch classes, researchers are revealing surprising capabilities of bacterial noncoding RNAs (ncRNAs). Natural riboswitches can form intricate structures to perform essential molecular sensing and genetic switching functions, usually without the assistance of protein factors. In most instances, a single aptamer domain partners with an expression platform domain, wherein the latter usually can form multiple structural states whose conformation dictates gene expression levels [6,15]. Some of the complex features of riboswitches have been revealed by studies that establish the atomic-resolution structural models for riboswitch aptamers [16,17], or that examine the mechanisms of RNA switching using single-molecule biophysics (e.g [18–20]) or NMR (e.g [21,22]) studies. Given what is already known about riboswitches and the promising landscape for new riboswitch discoveries, the rich diversity of functions possible for these RNA-based gene control devices is likely much greater than is currently understood. Furthermore, as the details of extant riboswitch functions and mechanisms are explored, we gain insight into the possible functions of ancient riboswitches [15] that were probably abundant during the RNA World – a proposed era in early evolution that preceded the evolutionary emergence of DNA and proteins [23,24].

**Figure 1.**
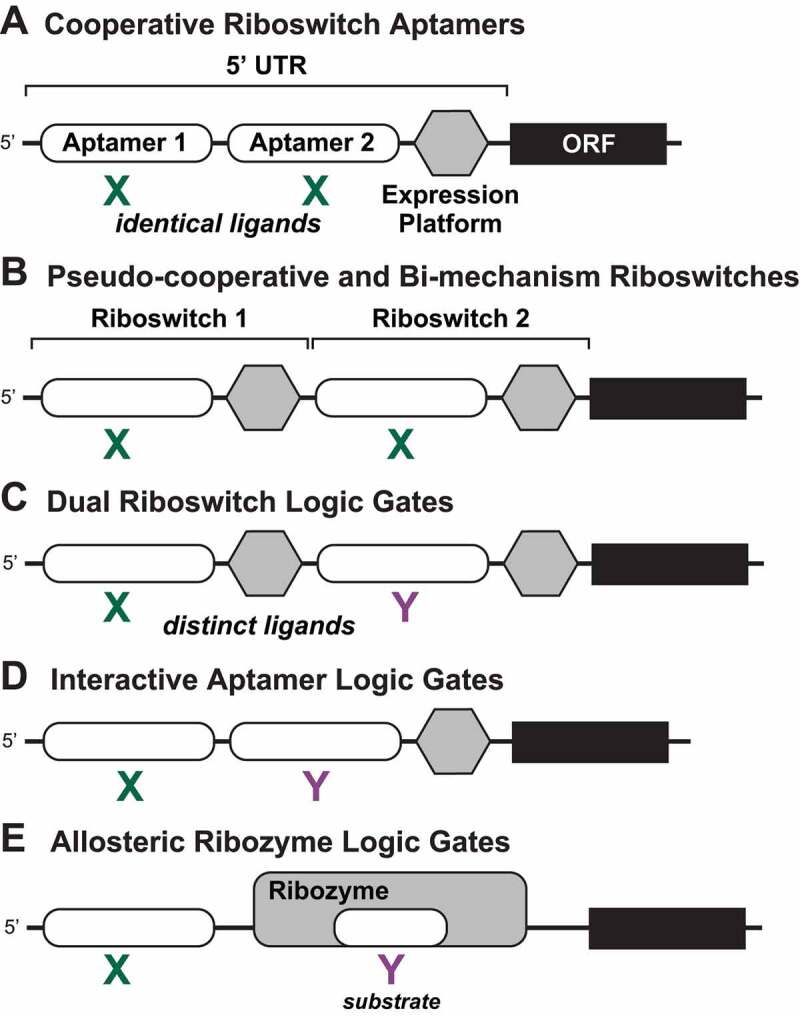
**Previously known tandem architectures for riboswitches and their established functions**. (A) Cooperative riboswitch aptamers carry highly similar aptamer domains that bind chemically identical ligands and associate with a single expression platform. Examples of this riboswitch architecture demonstrate cooperative ligand binding and a steeper dose-response curve [27]. (B) Pseudo-cooperative [27] and bi-mechanism [55] riboswitches involve the tandem arrangement of independently functioning riboswitches that respond to chemically identical ligands. For a bi-mechanism system, each riboswitch operates with a different regulatory mechanism (e.g. one transcriptional and one translational). (C) Dual riboswitch logic gates [25] involve the tandem arrangement of independently functioning riboswitches that respond to different target ligands, here depicted as X and Y. (D) Interactive aptamer logic gates [56] are formed by two adjacent aptamers that respond to different target ligands and associate with a single expression platform. Ligand binding by one aptamer affects the function of the adjacent aptamer. (E) Allosteric ribozyme logic gates [59] involve allosteric regulation by an aptamer for the function of a ribozyme that requires a second distinct compound for its activity.

Most studies to date have focused on riboswitches composed of a single ligand-sensing aptamer domain that works in concert with a single, downstream expression platform that interfaces with the cellular machinery used to transcribe or translate the information stored in DNA. However, an intriguing adaptation of some modern riboswitches is the tandem assembly of aptamers or of complete riboswitches to produce RNA systems that exhibit more intricate gene control functions [25,26] (Figure. 1). Tandem arrangements were originally encountered with glycine riboswitches that, unlike most riboswitch arrangements, commonly contain two similar aptamer domains with a single expression platform [27]. Several types of tandem riboswitches that function independently also have been identified. Sometimes, these include multiple instances of the same riboswitch class, creating multiple checkpoints for assessing the cellular availability of the cognate ligand along the transcription and/or translation process to decide whether the encoded gene should be expressed.

**Figure 2.**
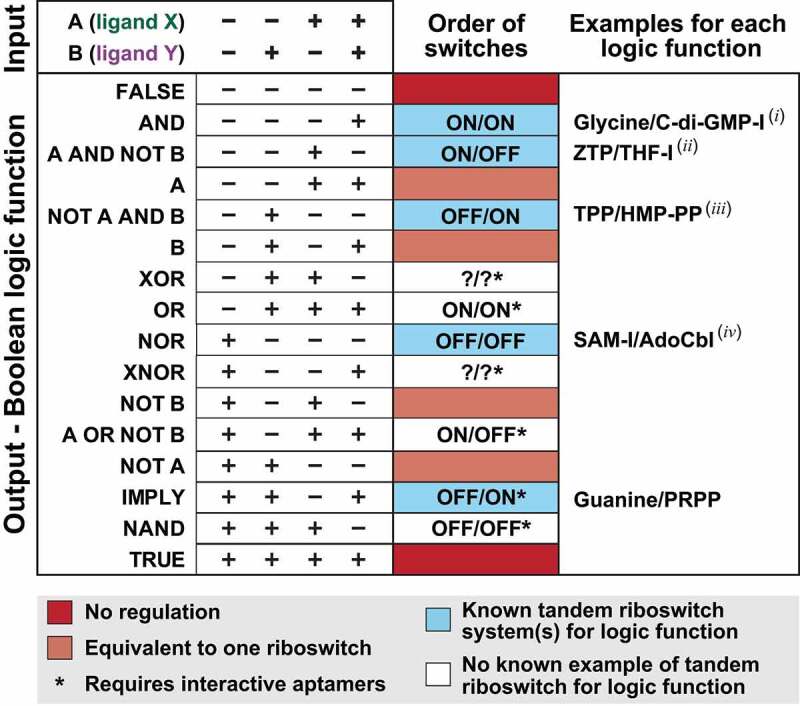
**All theoretical two input Boolean logic gate systems and their relationship to riboswitch gene control systems**. Depicted are the truth tables [28] for all possible gene regulation logic gate systems based on the presence (+) or absence (−) of ‘inputs’ A and B, where A represents the cognate ligand of the first RNA domain and B represents the cognate ligand for the second domain (e.g. ligands X and Y as presented in Figure. 1). There are four possible combinations of ‘inputs’ (presence or absence of A or B occupying the aptamer binding pocket) and 16 possible gene expression ‘outputs’, i.e. whether expression of the downstream gene is on (+) or off (−). Each output is named for its corresponding Boolean logic function [28] and is coloured to reflect its known or possible riboswitch manifestation. Note that ‘FALSE’ and ‘TRUE’ outputs (dark red) are not switches, and therefore have no utility for gene control. ‘A’, ‘B’, ‘NOT A’, and ‘NOT B’ outputs (light red) represent gene control outcomes that are identical to those achievable by single riboswitches with a single ligand input, and therefore tandem arrangements are unnecessary. Natural examples of five of the remaining ten genetically practical two-input Boolean logic gate functions are known to exist (blue). White boxes indicate that a tandem, two-input riboswitch system has not yet been observed in nature. Question marks indicate that it is not apparent how tandem riboswitches would function to create XOR and XNOR logic gates. Published examples are presented for the five known logic gates as follows: C-di-GMP-II/Group I Rz [59], ZTP/THF [89], TPP/HMP-PP [105], SAM-I/AdoCbl [25], and Guanine/PRPP [56]. Additional examples include (*I*) T box^Leu^/ppGpp [56] and glycine/c-di-GMP (this study); (*ii*) ZTP/glycine (this study), Na^+^-I/c-di-AMP [118], and SAM-I/T box^Met^ (this study); (*iii*) T box^Met^/SAM-I (this study), AdoCbl/C-di-GMP-I (this study), AdoCbl/C-AMP-GMP (this study), Adenine/T box^Tyr^ (this study), Glycine/T box^Ser^ (this study) and AdoCbl/T box^Val^ (this study); (*iv*) AdoCbl/SAM-I (this study) (Table 2).

**Figure 3.**
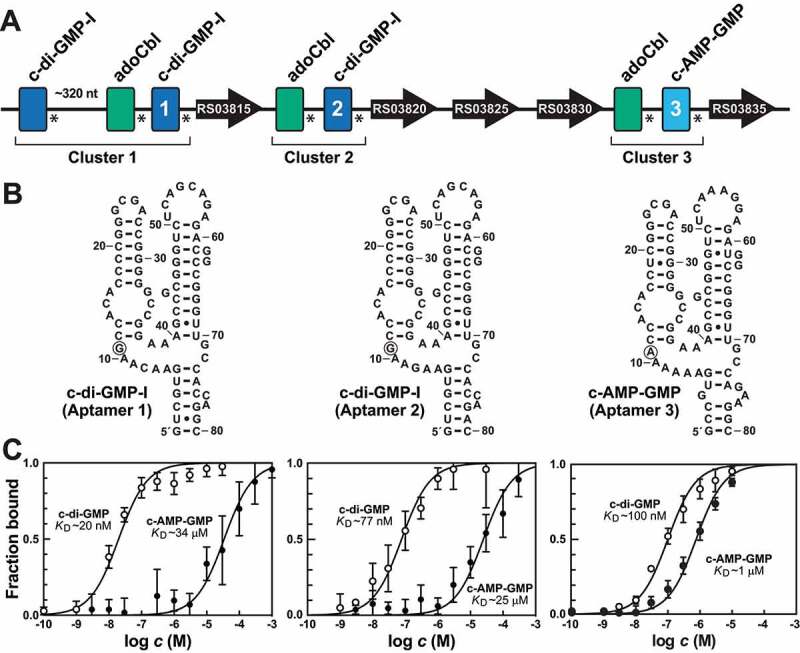
**An unusual series of tandem riboswitches that sense AdoCbl, c-di-GMP, and c-AMP-GMP ligands**. (A) Schematic representation of seven riboswitches occurring within a seven kb region of the genome of the bacterium *D. acetoxidans*. Asterisks denote the locations of putative intrinsic transcription terminators, indicating each aptamer functions as an independent riboswitch. Aptamers labelled 1, 2, and 3 were subjected to ligand binding assays as depicted in B. (B) Sequences and secondary structure models for aptamers evaluated by in-line probing assays. Circled nucleotides identify a position directly contacting the ligand that is known to be important for the selective binding of c-di-GMP (G nucleotide) or c-AMP-GMP (A nucleotide) [43]. (C) Binding curves resulting from in-line probing assays conducted on aptamers 1 through 3 (left to right) with c-di-GMP and c-AMP-GMP. Data points represent the average modulation at three locations, where the error bars represent the standard deviation at each concentration tested.

In other instances, two riboswitches occur in tandem within the same 5ʹ untranslated region (5ʹ UTR) that respond to different ligand molecules, creating a more sophisticated regulatory system. In these cases, tandem riboswitch systems are analogous to two-input logic gates, wherein two inputs (i.e. the two different ligand molecules) contribute to a single decision (i.e. whether the gene is expressed). In mathematics and computing, the possible combinations of inputs and outputs for two-input logic gates can be described by Boolean functions (Figure. 2) [28]. Likewise heterogeneous tandem riboswitches can usually be described in terms of the corresponding Boolean logic gate created, which usually depends on several factors such as whether each riboswitch is an ‘ON’ switch (ligand binding activates gene expression) or an ‘OFF’ switch (ligand binding suppresses gene expression), the linear order of the riboswitches in the RNA, and whether their aptamers functionally interact with one another. The first two-input Boolean logic gate to be described consisted of a riboswitch for *S*-adenosylmethionine (SAM) followed by a riboswitch for coenzyme B_12_ (adenosylcobalamin or AdoCbl) [25]. Specifically, both riboswitches are OFF switches and the binding of either SAM or AdoCbl to their corresponding riboswitch turns off gene expression, thus each RNA transcript functions as a NOR logic gate (Figure. 3) [28].

**Table 1.** Riboswitches and tandem riboswitch arrangements that respond to multiple, identical ligands.

One Expression Platform	Multiple Expression Platforms
**Single Aptamer**	**Pseudo-cooperative**
THF-I [44]	TPP [25,54]
C-di-AMP [45–47]	AdoCbl [25]
PreQ_1_-I (type 1) [48]	SAM-I [This study] SAM-II [This study] Moco [10]
Mg^2+^-I [49]	Wco [10]
NiCo [50]	FMN [This study]
**Dual Aptamer**	C-di-AMP [This study]
Glycine [27]	C-di-GMP-I [42,93]
Guanidine-II [70]	C-di-GMP-II [This study]
	Lysine [This study]
	Li^+^-I [100]
	Mg^2+^-I [25]
	Glutamine-I [100] Glycine [This study] Guanine [This study] ZTP [This study]
	**Bi-mechanism**
	SAM-II/SAM-V [55]
	SAM-II/SAM-I/-IV [This study]
	SAM-I/-IV/SAM-II [This study]

**Table 2.** **Tandem RNA systems that form Boolean logic gates to regulate gene expression**. *Several distinct sequence variants of the c-di-GMP-II/Group I ribozyme arrangement exist [59], but they occur exclusively in isolates of *C. difficile* and thus are counted only once.

Position		Boolean Logic	Count	Citation
**1**	**2**			
ppGpp	T box^Leu^	AND	15	[56]
T box^Leu^	ppGpp	AND	16	[56]
Glycine	C-di-GMP-I	AND	6	[This study]
C-di-GMP-II	Group I Rz.	AND	1*	[59]
SAM-I	AdoCbl	NOR	8	[25]
AdoCbl	SAM-I	NOR	3	[This study]
ZTP	THF-I	A AND NOT B	10	[89]
ZTP	Glycine	A AND NOT B	5	[This study]
Na^+^-I	C-di-AMP	A AND NOT B	5	[118]
T box^Met^	SAM-I	A AND NOT B	2	[This study]
SAM-I	T box^Met^	NOT A AND B	7	[This study]
TPP	HMP-PP	NOT A AND B	131	[105]
AdoCbl	C-di-GMP-I	NOT A AND B	2	[This study]
AdoCbl	C-AMP-GMP	NOT A AND B	1	[This study]
Adenine	T box^Tyr^	NOT A AND B	5	[This study]
Glycine	T box^Ser^	NOT A AND B	2	[This study]
AdoCbl	T box^Val^	NOT A AND B	2	[This study]
Guanine	PRPP	IMPLY	192	[56]

For the current study, we used computational methods to systematically search bacterial genomic sequence databases for additional examples of tandem riboswitch arrangements. In total, 24 different riboswitch classes appear in tandem either with their own class, with another riboswitch class, or with another regulatory RNA. Nine different two-input Boolean logic gates formed by RNA are reported that have not previously been described. Our findings reveal that some bacterial species use tandem riboswitches to make sophisticated gene control decisions that otherwise would require the involvement of protein factors. These findings also support the view that ancient organisms of the RNA World likely exploited complex riboswitch architectures to carry out advanced regulatory functions long before proteins arose in evolution.

## Materials and methods

### RNA comparative sequence analyses

Identification of riboswitch and ribozyme representatives from experimentally validated classes was achieved by homology searches using Infernal version 1.1 [29]. Databases searched included RefSeq [30,31] version 80 (containing 44,270 named bacterial species) and additional environmental microbial sequences as previously described [32,33]. Protein coding regions were annotated using the Conserved Domain Database [34] version 2.25 as previously described [35]. RNA domains were annotated using the Rfam database version 12.2 [36], WU-BLAST [37], and RAVENNA [38] global searches using published riboswitch alignments as previously described [35]. Genomic locations and the upstream and downstream genetic contexts for each riboswitch or ribozyme representative were tabulated, sorted, and manually examined. Tandem ncRNA domains were judged to exist if no intervening ORFs were observed.

Manual assessments of the representatives identified by computation means were then made. Some pseudo-cooperative riboswitch candidates were recognized as false positives because the genomic coordinates of the two riboswitches were overlapping, and thus represent a single riboswitch. Some examples of pseudo-cooperative riboswitches identified in short environmental (metagenomic) DNA sequencing reads that lacked any genetic context beyond the riboswitch sequences were also discarded because the validity of these examples could not be further verified. Other potential tandem riboswitch arrangements were discarded if the sequencing, assembly, or annotation of the DNA sequences cast doubt on the proposed architecture. For example, cases wherein large gaps (defined as more than 300 nucleotides of intervening sequence) between the end of one riboswitch aptamer and the beginning of the next were excluded. Long gaps are often indicative of an unannotated intervening ORF, which would likely separate the RNA structures and functions. Also discarded were possible tandem riboswitches that are separated by stretches of numerous undefined nucleotide positions, as indicated by repetitive N letters in the sequence database.

Some false positive candidates also could have been retained in our dataset. These misassigned tandem arrangements could be due to DNA sequence assembly errors in the databases that result in duplicated sequences, or annotation errors that fail to identify ORFs that would otherwise separate riboswitches.

When possible, proposed Boolean logic function assignments for each tandem system were made by predicting whether each riboswitch serves as an ON or OFF switch by examining the expression platform, and then evaluating the theoretical truth table that would result.

### Chemicals

All chemicals and chemically synthesized DNA oligonucleotides were purchased from Sigma-Aldrich unless otherwise noted. [γ-^32^P]-ATP was purchased from PerkinElmer. Enzymes were purchased from New England Biolabs unless otherwise noted.

### In-line probing assays

RNA oligonucleotides were prepared as previously described [39]. Briefly, RNA oligonucleotides were synthesized *in vitro* using laboratory-prepared T7 RNA polymerase from a synthetic DNA oligonucleotide template. The resulting RNA transcripts were purified by denaturing (8 M urea) 10% polyacrylamide gel electrophoresis (PAGE) and recovered from the gel via crush-soak treatment in 10 mM HEPES buffer (pH 7.5 at ~20°C), 200 mM NaCl, and 1 mM ethylenediaminetetraacetate (EDTA) followed by precipitation with ethanol. Purified RNA was dephosphorylated with rAPid alkaline phosphatase (Roche Applied Science). Dephosphorylated RNA was 5′ ^32^P-radiolabeled with [γ-^32^P]-ATP by using T4 polynucleotide kinase following the manufacturer’s directions. The radiolabeled RNA was purified as described above and subjected to in-line probing analysis.

In-line probing assays were performed as previously described [40,41]. Briefly, trace amounts of 5′ ^32^P-radiolabeled RNA was incubated in the presence or absence of ligand candidates (c-di-GMP or c-AMP-GMP) at 28°C for 42 to 48 h. Each 10 µL reaction contained 20 mM MgCl_2_, 100 mM KCl, and 50 mM Tris-HCl (pH 8.3 at ~20°C). Spontaneous RNA cleavage products were separated via denaturing 10% PAGE. The resulting gels were dried and the cleavage patterns visualized and quantified using a phosphorimager (GE Healthcare Life Sciences).

Dissociation constants were established by increasing the concentration of the appropriate ligand and quantifying the band intensities at nucleotides which exhibit variation in intensity using ImageQuant (GE Healthcare Life Sciences). These intensities were normalized to a non-modulating band and the resulting values were averaged to calculate a ‘fraction bound’, which was scaled between 0 and 1. The scaled values were plotted against the logarithm of the molar concentration of the ligand, using GraphPad Prism 7. In cases where this fraction bound did not reach saturation, the maximum scaling value was extrapolated from the data by assuming 1:1 binding, which is observed for c-di-GMP-I riboswitches examined biochemically [42] and by x-ray crystallography [43]. The apparent dissociation constant (*K*_D_) was then determined by fitting the data to a sigmoidal dose-response curve with a Hill coefficient of 1.

## Results and discussion

### Known mechanisms for tandem riboswitch systems

Below we summarize the previously established biochemical and biological functions of the different types of tandem riboswitch systems (Figure. 1). This provides a framework for categorizing and predicting the functions of any novel riboswitch architectures discovered in the future. We also propose new nomenclature and provide brief descriptions of previously reported natural tandem riboswitch systems corresponding to each type of architecture.

#### Cooperative riboswitch aptamers

Several riboswitch classes are known to use a single aptamer architecture to cooperatively bind two [44–48] or more [49,50] of its cognate ligand using distinct binding pockets. However, for the current discussion, we are only considering separate, tandem aptamer arrangements that exhibit cooperative ligand binding. In their simplest tandem form, cooperative riboswitch aptamers employ two homologous aptamer domains that reside immediately adjacent to each other, followed by a single expression platform (Figure. 1A). Because the aptamers sense ligands that are identical in chemical structure, the most obvious biochemical function for this arrangement is for the aptamers to work cooperatively such that ligand binding in one aptamer improves the affinity of ligand binding in the other aptamer. Otherwise, if the aptamers were to work independently of each other and together add no functional value, then the tandem aptamer arrangement would be redundant and presumably would disappear through normal evolutionary processes.

A tandem aptamer, single expression platform architecture is commonly observed for glycine riboswitches, where the arrangement is necessary for the observed steep binding and transcription termination curves in vitro, and for gene regulation in cells [27]. Such composite riboswitch devices exhibit a Hill coefficient for ligand binding that is greater than 1 [27,51–53], which enables changes in gene expression to occur over a narrower range of ligand concentration changes. There has been some disagreement about the natural performance characteristics of glycine riboswitches with tandem aptamers, which we discuss in some detail later.

#### Pseudo-cooperative and bi-mechanism riboswitches

Tandem riboswitches that function independently but respond to identical ligands (Figure. 1B) can have two arrangements that exhibit slightly different biological outcomes. The first arrangement, called ‘pseudo-cooperative’ riboswitches, generates a steeper dose-response curve, much like that observed for the homologous cooperative aptamer architectures, as noted above. In natural examples, such as that seen with tandem riboswitches for thiamin pyrophosphate (TPP) [54], the first representative can either terminate transcription or allow readthrough. This first riboswitch responds to its cognate ligand according to a normal dose-response curve as expected for a 1:1 interaction between a ligand and its aptamer. However, the fraction of RNA transcripts that experience transcriptional readthrough (continuing on to encounter the second riboswitch in the tandem arrangement) will undergo a secondary decision point between transcription termination or readthrough. If the riboswitches are similarly sensitive to the ligand’s concentration, this tandem arrangement yields a steeper or more ‘digital’ dose-response curve at the level of gene expression. Although the aptamers are not truly cooperative, in that binding at one riboswitch does not influence binding at the other riboswitch, this ‘pseudo-cooperative’ functional outcome is expected to be similar to that for riboswitches with homologous cooperative aptamers [54].

The second possible arrangement, produced using ‘bi-mechanism’ riboswitches, regulates gene expression at two different mechanistic levels, as is observed for tandem SAM-II/SAM-V riboswitches [55]. In each tandem representative, the initial SAM-II riboswitch is predicted to terminate transcription upon ligand binding, whereas the subsequent SAM-V riboswitch is predicted to directly regulate translation initiation. Again, if the kinetics of ligand modulation are similar between the two riboswitches, a pseudo-cooperative dose-response curve for gene expression will result. In addition, and perhaps more importantly, tandem riboswitch systems that operate at two distinct regulatory levels could more efficiently control the expression of their associated open reading frames (ORFs) by adding a temporal component to their regulation. Simple riboswitches that regulate transcription termination avoid production of unnecessary mRNA transcripts in response to the concentration of their cognate ligand. However, bi-mechanism riboswitches might offer even greater efficiency by using the second riboswitch to regulate the expression of the resulting full-length mRNA. The second riboswitch retains the ability to turn off gene expression by preventing translation initiation at a later timepoint if ligand availability increases [55], which is expected to be more efficient than simple mRNA degradation and resynthesis.

#### Dual riboswitch logic gates

The occurrence of two distinct riboswitch classes in series (Figure. 1C) creates a tandem system that responds to two different ligands to yield various genetic outcomes, which parallel the ‘truth tables’ (accounting of signal inputs and expected outcomes) of some Boolean two-input logic gates [28] (Figure. 2). Both the order in which the riboswitch classes appear and the directionality of the riboswitch (ON or OFF switch) affect the truth table (describing the conditions under which gene expression is on or off) that results under the various ligand states. For example, the first two-input tandem riboswitch system to be reported [25] employs a SAM-I riboswitch followed by a riboswitch for AdoCbl. Each individual riboswitch functions as a genetic OFF switch, and therefore high concentrations of either ligand (or both) suppress transcription of the associated ORF. The resulting truth table entry represents a NOR gate (Figure. 2).

#### Interactive aptamer logic gates

Riboswitches with multiple aptamers that bind chemically distinct ligands but associate with a single expression platform (Figure. 1D) also can function as two-input Boolean logic gates. Examples of this tandem arrangement were found that respond to the nucleobase guanine and the activated ribose molecule 5-phosphoribosyl-1-pyrophosphate (PRPP) [56]. The PRPP aptamer interferes with the formation of an intrinsic terminator stem to activate gene expression when its ligand is bound. Intrinsic terminator stem formation is known to cause RNA polymerase to cease transcription [57,58], whereas aptamer interference with this structure can favour production of the complete mRNA [2–9]. However, guanine binding to the first aptamer is predicted to weaken the affinity that the PRPP aptamer exhibits for its ligand, and vice versa. Thus, the two ligands work in opposition to regulate gene expression in a manner that creates an IMPLY [28] Boolean logic gate (Fig. 2). Although building logic gates by arranging two independently functioning riboswitches in tandem might be evolutionarily simpler, a broader collection of logic functions can be created by employing aptamers that have evolved to directly affect each other’s ligand binding functions.

#### Allosteric ribozyme logic gates

An exceptionally rare arrangement between an aptamer for the bacterial signalling molecule c-di-GMP and a self-splicing ribozyme triggered by a GTP nucleotide has been observed to function as a two-input Boolean AND gate [59,60]. This riboswitch architecture is unusual in that the expression platform is a group I self-splicing ribozyme, and these RNA enzymes are known to use guanosine or any of its 5́-phosphorylated derivatives to initiate the splicing process [45]. This metabolite-dependent ribozyme is allosterically controlled by a c-di-GMP-II riboswitch aptamer, such that both small molecules are required for gene expression to occur [59,61,62]. Tandem arrangements between riboswitch aptamers and ribozymes thus allow surprisingly complicated gene control devices to be assembled from RNA.

### The search for additional tandem aptamer and riboswitch representatives

Most known riboswitch and ribozyme classes exhibit extensive nucleotide sequence and secondary structure conservation. Therefore, homology search algorithms that evaluate nucleotide sequence conservation and covariation can be used to identify additional representatives in available genomic sequence databases (e.g [35,61,63].). Once identified, their genomic locations can be recorded and compared with the locations of other known structured ncRNA representatives. RNA motif representatives that reside within the same 5ʹ untranslated region can then be evaluated as possible tandemly arranged RNA devices.

We conducted extensive searches within 44,270 different bacterial genomes in RefSeq 80 and environmental DNA samples (See MATERIALS AND METHODS) for additional representatives of all validated riboswitch aptamer classes and most self-cleaving ribozyme classes using the homology search algorithm Infernal [29]. The genomic location of each riboswitch or ribozyme representative was recorded, and the resulting collection of genomic locations was used to identify instances where two or more ncRNA representatives reside in tandem. For the current study, we only pursued representatives that were found immediately adjacent to and oriented in the same direction as another known structured or regulatory RNA for further analysis. However, some major ncRNAs such as ribosomal RNAs (rRNAs) and transfer RNAs (tRNAs) were excluded from our analyses.

### Tandem aptamer arrangements

Our analyses revealed additional representatives of previously known tandem aptamer and riboswitch arrangements, as well as several surprising novel arrangements (Table 1). In this and subsequent sections, all tandem architectures encountered in our analyses are summarized in a manner generally organized based on the riboswitch classes involved. We focus first on RNAs that carry more than one aptamer domain associated with a single expression platform.

#### Tandem glycine aptamers

The first tandem aptamer architecture to be identified for riboswitches involved glycine-binding RNAs that naturally positioned two highly similar aptamer domains upstream of a single expression platform [27]. By evaluating this architecture, it was hypothesized that the system would likely function to bind two glycine molecules cooperatively. This proposed function provided the simplest explanation for why a riboswitch would arrange two near-identical aptamer domains that regulate gene expression through a single expression platform. Alternatively, it was possible that the aptamers would bind different ligands, although this seemed unlikely given the strong conservation of the nucleotides between the aptamers. Indeed, initial experiments [27,51,52] and subsequent structural analyses [53] demonstrated that the aptamers can function cooperatively.

To achieve cooperative function, riboswitches of this type must exploit ligand binding in one aptamer in a manner that promotes a structural configuration of the remaining aptamer such that ligand binding is favoured or somehow reinforced. This indeed appears to be the case for tandem glycine aptamers, wherein the two RNA domains contact each other in the ligand-bound state [52,53], presumably to either pre-organize an aptamer to receive the ligand or to reduce the probability that the aptamers will release their ligands once bound. Whereas the majority of glycine riboswitches comprise two glycine-binding aptamers, there are also ‘singlet’ glycine riboswitches that only contain one ligand-binding aptamer [64]. In cases where only one full glycine aptamer is present, it is often adjacent to a ‘ghost aptamer’, which does not bind glycine but supports function of the intact aptamer [64,65].

Although the cooperative nature of tandem glycine riboswitch aptamers has been disputed [66,67], the data supporting this alternative view was generated under non-kinetically controlled conditions. In other words, the genetic switch might function cooperatively during the very brief time that it takes RNA polymerase to progress from the point at which the aptamers emerge from the enzyme to the point at which the polymerase moves past the terminator stem. Perhaps natural tandem glycine aptamer systems are cooperative, but that under certain equilibrium conditions this cooperativity is masked [68]. Indeed, in cells it appears that ligand binding to both aptamers is essential for glycine-mediated gene expression because mutations to either aptamer completely disrupt regulation [27], which is a result expected for cooperative aptamer systems. Furthermore, no plausible mechanism for tandem riboswitch aptamers has been proposed that does not involve the influence of binding characteristics by one aptamer on its neighbour.

Intriguingly, certain glycine riboswitch variants have been observed that carry mutations in the ligand-binding cores of either one or both aptamers [69]. These RNAs have been shown to reject glycine, and therefore have likely changed their ligand-binding specificity. Thus, it is likely that additional interactive aptamer logic gates and cooperative riboswitch classes are formed from the same general architecture used by the widespread tandem glycine aptamer systems.

#### Tandem guanidine-II aptamers

One of the smallest and simplest known tandem aptamer systems is represented by guanidine-II riboswitches [70]. Two guanidine binding pockets are formed by the interaction of the four-nucleotide loops of two highly similar hairpin structures [71,72]. Remarkably, this simple architecture forms binding pockets that are both selective for guanidine and strongly cooperative. Cooperativity is achieved because the formation of each ligand-binding pocket with docked ligand requires the presence of the other hairpin.

Our search for tandem arrangements of guanidine-II riboswitches with other riboswitch or ribozyme classes failed to reveal any examples. This is not surprising because we have not observed tandem arrangements with any of the four known guanidine riboswitch classes [70,73–76], suggesting that cells do not have a need to integrate information on guanidine concentration with any other chemical signal.

Furthermore, it is known that a single hairpin matching the consensus for guanidine-II aptamers cannot function well, as demonstrated by mutations in the conserved nucleotides of the partner hairpin [70]. Therefore, it is also unlikely that singlet guanidine-II aptamer arrangements without additional structural support will be found. Unfortunately, given the simplicity of the consensus sequence and structure of each hairpin and the likely overwhelming abundance of false positive examples, we cannot accurately search for singlet guanidine-II aptamer arrangements. Thus, we have not ruled out the possibility that a functional guanidine-II aptamer could assemble with another aptamer to create mixed tandem architectures.

#### Tandem aptamers of NAD^+^-I riboswitches

Riboswitches of the NAD^+^-I class always employ two highly similar aptamers to regulate gene expression in response to changing levels of the ubiquitous coenzyme nicotinamide adenine dinucleotide (NAD^+^) or one of its close derivatives [77]. The aptamers are followed by a single expression platform, suggesting that the aptamers might bind identical ligands in a cooperative fashion as observed for many glycine riboswitches. Strangely, the two domains have resisted attempts to demonstrate selective binding of the nicotinamide moiety of NAD^+^ or other related compounds in vitro. Rather, the first domain functions as a precise sensor for the ADP moiety of the coenzyme [77–79]. The second domain can bind the ADP moiety at high concentrations [79], but it is not certain that this interaction is biologically relevant. Indeed, another study has also demonstrated binding of ATP at high concentrations for other riboswitch classes [80], but this is unlikely to represent the natural ligand [81].

It has been proposed that domain 1 of NAD^+^-I riboswitches naturally recognizes ADP and that the highly similar domain 2 recognizes the pyrophosphorylated nicotinamide mononucleotide (NMN) moiety [77]. The potential molecular recognition contacts on the ADP moiety are very similar to those on the pyrophosphorylated NMN moiety. In vitro binding assays might have failed because a critical co-ligand for this second aptamer (such as a metal cation, a second small molecule, or a protein factor) is absent outside its natural cellular environment. If this hypothesis is correct, then this unusual tandem aptamer system senses only a single molecule by exploiting similar aptamers to selectively bind similar chemical moieties. Regardless of the precise structure and function of the tandem aptamers in NAD^+^-I riboswitches, we did not observe examples of this riboswitch class residing near other riboswitches or ribozymes.

### Tandem pseudo-cooperative riboswitches

Several tandem riboswitch arrangements involve two or more complete riboswitches (aptamer plus expression platform) sequentially arranged upstream of a single ORF that sense identical ligands. Most commonly, these arrangements are formed from riboswitch representatives of the same structural class. Below we describe various examples of tandem riboswitches for the same ligand that are predicted to function with pseudo-cooperative characteristics.

#### Tandem TPP riboswitches

TPP riboswitches [2,3] sense the activated form of thiamin (vitamin B_1_), an essential coenzyme. It is the most abundant riboswitch class known, with representatives present in organisms from all domains of life [6]. Thus, there are many opportunities for TPP riboswitches to be identified in tandem arrangements. Indeed, we identified a total of 213 representatives generally grouped into the TPP riboswitch class that reside in tandem with another member of the same class, including one example of three consecutive TPP riboswitches.

Although many of these are likely to be pseudo-cooperative systems, we have determined that some RNAs currently classified as TPP riboswitches carry sequence variations that alter their ligand specificity to favour thiamin or thiamine monophosphate [82]. Therefore, it is not yet clear whether some of these arrangements might sense TPP and one of its close metabolic precursors to function differently. Sorting through the precise ligand specificities for the tandem TPP riboswitch systems will be necessary to determine the precise functions of each.

#### Tandem glycine riboswitches

Our new computational searches revealed examples of glycine aptamers occurring in triplets (two examples) or quadruplets (16 examples). In both examples of triplets, each of the three aptamers represents a ‘type-1 singlet’ [64]. Specifically, this architecture involves a single glycine aptamer followed by a ghost aptamer [64,65] and an expression platform, and this is repeated three times. Each repeat likely functions as a simple riboswitch that generates a conventional 1:1 ligand binding profile. When combined, they presumably function as a pseudo-cooperative riboswitch system.

The quadruplet glycine aptamer arrangements are formed by the tandem assembly of two glycine riboswitches that each carry two aptamers. Thus, we predict that the quadruplet aptamer examples combine the performance characteristics of the cooperative tandem aptamer arrangement common for most examples within this class with the pseudo-cooperative characteristics of tandem riboswitches that sense identical ligands. Presumably, stacking two riboswitches that are inherently cooperative in the same mRNA blends two mechanisms for regulating gene expression over a narrower glycine concentration range than would be possible by using either strategy alone.

#### Tandem SAM-I riboswitches

SAM-I riboswitches [83–85] represent another common class, and thus offer many opportunities for tandem arrangements. Indeed, there are 164 examples of double SAM-I riboswitches and one example of triple SAM-I riboswitches. These examples are likely to function as pseudo-cooperative systems, although other bi-mechanism tandem riboswitches involving SAM-I RNAs are discussed in greater detail below.

#### Tandem SAM-II riboswitches

We identified 44 examples of tandem SAM-II [86] riboswitches. In some instances, each SAM-II aptamer is associated with an intrinsic terminator stem, suggesting that they function as pseudo-cooperative systems. However, some have mixed expression platforms, where the first aptamer precedes a terminator stem and the second appears to regulate access to the ribosome binding site (RBS) of the adjacent ORF. Regulation of ribosome binding by using anti-RBS regions (sequences complementary to the purine-rich RBS) is a common mechanism by which riboswitches regulate gene expression [6,87,88]. Arrangements involving riboswitches that use transcription termination and translation initiation mechanisms to regulate a single ORF should permit more complex gene control outcomes as previously proposed [55] and as discussed above. Such bi-mechanism systems are further discussed in a later section for arrangements involving SAM-II riboswitches in tandem with other structurally distinct SAM riboswitch class members.

#### Tandem ZTP riboswitches

ZTP riboswitches [89] sense various phosphorylated derivatives of the purine biosynthetic intermediate AICAR, which serve as alarmone signals when they accumulate during coenzyme tetrahydrofolate (THF) starvation [90]. The AICAR derivative carrying three phosphates (ZTP) predominates when there is insufficient 10-formyl THF to promote the completion of the nascent purine ring of AICAR to eventually generate ATP and GTP. Thus, ZTP riboswitches typically turn on the expression of folate biosynthesis genes when 10-formyTHF levels are deficient and ZTP levels increase.

Five instances of tandem ZTP riboswitches were identified in our analyses, and four of these are associated with genes associated with THF metabolism where they are predicted to activate expression when ZTP is elevated. The remaining tandem representative is associated with the gene *moeA*, which codes for a protein that promotes the addition of molybdate into molybdopterin as the final step of molybdenum cofactor (Moco) biosynthesis [91]. Both ZTP aptamers in this latter system are predicted to use terminator stems to turn off gene expression when ZTP is bound. Therefore, even just among these few examples, there are both tandem ON and tandem OFF ZTP riboswitch systems. This unusual gene association and unusual directionality for gene control by ZTP riboswitches might be due to the fact that both THF and Moco are biosynthesized using a pterin ring system derived from GTP. Perhaps in the host species, cells choose to prioritize the biosynthesis of THF over Moco when pterin levels are reduced and ZTP concentrations are elevated.

#### Tandem c-di-GMP-I riboswitches

The bacterial second messenger c-di-GMP is used by many bacteria to regulate major physiological changes in cells [92]. Remarkably, 347 doublet and 12 triplet arrangements of class I c-di-GMP riboswitches (called c-di-GMP-I) [42,59] were identified. A previous report [93] describes the confirmed function of a triplet c-di-GMP-I system from a strain of *Bacillus thuringiensis*, which exhibits more digital gene expression characteristics as expected (see above). The number of unique tandem doublet and triplet c-di-GMP-I riboswitches in this dataset is higher than for any other riboswitch class. Perhaps this is due to the fact that the lifestyle changes regulated by these riboswitches are binary, and thus might best be fully triggered by small changes in ligand concentration rather than altered gradually as enabled by a single riboswitch system.

#### Tandem c-di-AMP riboswitches

The aptamer domain of c-di-AMP riboswitches [94] is already known to bind two c-di-AMP molecules using distinct binding sites [45–47]. If each c-di-AMP molecule is bound by an aptamer in a cooperative fashion, then tandem arrangements of riboswitches would create unusual cooperative/pseudo-cooperative riboswitch arrangements. Indeed, we identified 77 c-di-AMP riboswitch doublets, wherein most appeared to carry an intrinsic terminator between the two aptamer domains as observed with other pseudo-cooperative systems. In some instances, the RNAs appear to lack a strong intrinsic terminator stem between the first and second aptamer domains. This presents an intriguing possibility that a cooperative, independently folding c-di-AMP aptamer that binds two c-di-AMP molecules might interact with another similar aptamer to bind four c-di-AMP molecules cooperatively. Given this intriguing possibility, the precise mechanisms used by these unusual tandem c-di-AMP riboswitch systems merits further investigation.

#### Many additional pseudo-cooperative riboswitches exist

In addition to the sampling of tandem riboswitch systems described above, there are many other tandem riboswitch arrangements that are also predicted to function with pseudo-cooperative characteristics. Specifically, we encountered the following tandem examples of riboswitches from the same class:

- c-di-GMP-II [59]: Numerous tandem c-di-GMP-II riboswitch arrangements also exist, and 59 doublets and 9 triplets, were observed in our study. Most of these appear upstream of a gene for putative surface anchor protein (COG4932) in various *Paraclostridium* species and other closely related bacteria, suggesting that the expression of this gene changes sharply with small chances in c-di-GMP concentration changes.
- Guanine [95]: A total of seven doublet and one triplet guanine riboswitch arrangements were identified in our analysis.
- Lysine [96,97]: 14 representatives of double lysine riboswitches were identified that appear to employ terminator stems as expression platforms.
- Moco [10]: Tandem riboswitches for the enzyme cofactor Moco reside upstream of biosynthesis genes for this coenzyme. Thirteen doublet and two triplet arrangements were observed.
- Wco [10]: Tandem riboswitches for tungsten cofactor (Wco) reside upstream of genes unrelated to tungsten cofactor biosynthesis, such as ferredoxin hydrogenase, nickel and zinc binding hydrogenase, and arsenic response proteins, which is consistent with the original proposal that RNA aptamers have become specialized to selectively respond to Moco or Wco [10].
- FMN [3,4]: A total of 31 doublet and four triplet FMN riboswitches were identified in our analysis.
- Mg^2+^-I [49,98]: Four representatives of tandem Mg^2+^-I riboswitches were identified that are predicted to activate gene expression when bound to ligand. Mg^2+^-I riboswitch aptamers are known to bind multiple Mg^2+^ ions cooperatively [49], and therefore the tandem riboswitch arrangements likely function as both cooperative and pseudo-cooperative systems like certain tandem arrangements of c-di-AMP riboswitches discussed above.
- Glutamine [99]: A substantial proportion of glutamine riboswitches occur in tandem to create pseudo-cooperative systems. In our current analysis, we identified 157 doublet and 4 triplet unique arrangements of glutamine riboswitches.
- AdoCbl [1]: A total of 26 doublets were identified in our analysis.
- Li^+^-I [100]: A total of 6 doublets and 1 triplet arrangements were observed.

### Tandem bi-mechanism riboswitches

As noted above, bi-mechanism systems might be employed simply to generate pseudo-cooperative gene control characteristics. However, functional value could also be gained by regulating gene expression via two different mechanisms, such as transcription termination and translation initiation. A bi-mechanism system exploiting these two gene control mechanisms would allow cells to regulate whether an mRNA is produced, as well as regulate whether the mRNA can be engaged by ribosomes to produce the encoded protein product. If the two gene control mechanisms are exploited with different time scales, then cells gain substantial gene regulation efficiency. For example, control of transcription termination offers a rapid way for cells to regulate how much of an mRNA is produced. In contrast, regulating ribosome access to pre-existing mRNAs would allow cells to retain metabolically expensive mRNAs for longer periods of time, and express their encoded protein products only when needed.

For many tandem riboswitch representatives, intrinsic transcription terminator stems are readily observed for the second riboswitch, indicating that the system is not of the bi-mechanism type. Whereas close proximity of the riboswitch aptamer to the start codon often indicates a translation control mechanism is used, the number of intervening nucleotides is not a perfect indicator of the expression platform used. Unfortunately, some examples exist where the expression platform cannot easily be predicted by sequence analysis alone. Thus, we have not comprehensively evaluated all tandem riboswitch arrangements to determine if they use the same regulatory mechanisms or if they function as bi-mechanism riboswitches. Below are described notable examples of bi-molecular systems, which are likely to be descriptive of others remaining undetected.

#### Bi-mechanism SAM-II and SAM-V riboswitches

As noted above, SAM-II riboswitches [86] are the second in a series of riboswitch classes reported to sense the coenzyme *S*-adenosylmethionine [6,39,101]. A few years later, a distinct-appearing collection of riboswitches called SAM-V were discovered [55]. Although the aptamer domains of SAM-II and SAM-V riboswitches have vaguely similar secondary and tertiary structure features and identically bind their target ligand [102,103], they often have unique phylogenetic and genomic locations, and they use distinct expression platforms [55,86].

Intriguingly, representatives of these two types of RNAs were commonly found in tandem, wherein SAM-II precedes SAM-V [55]. Among the original 120 tandem examples discovered, SAM-II riboswitches always precede an intrinsic terminator stem, whereas its downstream SAM-V partner always regulates translation by controlling access to the RBS. Although this bi-mechanism arrangement could allow pseudo-cooperative function, the tandem use of expression platforms that act first at the level of transcription and second at the level of translation provides an excellent opportunity for extreme conservation of energy. In the current analysis, we observed a total of 149 tandem arrangements of SAM-II and SAM-V riboswitches, which highlights the fact that some species are likely to gain much from exploiting bi-mechanism systems for regulating genes related to sulphur metabolism and SAM biosynthesis.

#### Other bi-mechanism tandem SAM-II riboswitches

As noted above, we identified 44 examples of doublet SAM-II riboswitches, wherein many carry expression platform arrangements like those observed for bi-mechanism SAM-II and SAM-V tandem riboswitches [55]. We also observed seven examples of a SAM-II riboswitch preceding a SAM-I/IV riboswitch [104], although these might function exclusively as pseudo-cooperative systems but use different aptamer classes to form the two riboswitches. Similarly, two examples were observed of the inverted configuration, wherein SAM-II resides after SAM-I/IV.

### Tandem arrangements of riboswitches that sense different ligands

By assembling riboswitches that sense different ligands in the same RNA transcript, evolution can readily create gene control systems that evaluate two or more chemical inputs to determine gene expression output. Several types of such riboswitch-based Boolean logic gates (Figure. 2) have been reported previously [25,56,59,89,105,106], and additional two-input riboswitch devices are likely to be exploited by modern cells. Although a total of 16 Boolean functions are possible for a two-input system [28], most easily visualized using truth tables, only ten of these would be reasonable options for regulation of gene expression. Natural examples of tandem riboswitches or similar ligand-binding RNAs represent five of the ten biologically practical logic gates (Figure. 2, blue-shaded outputs) have been reported. Below, we classify and summarize the genetic context and biological significance of 15 different arrangements of tandem RNA elements that form Boolean two-input logic gates (Table 2), nine of which were identified via the current analysis.

#### AND gates – ON/ON

An unusual tandem arrangement involves the co-localization of a c-di-GMP-II riboswitch aptamer and a group I self-splicing ribozyme [59,61]. These two RNA domains collaborate to form a Boolean AND gate [28], wherein both c-di-GMP and guanosine are required for maximum gene expression. Additional details regarding the interplay between the aptamer and the ribozyme are described in a later section.

An abundant two-input logic gate system comprised of a T box RNA specific for tRNA^Leu^ (herein called T box^Leu^) and a guanosine tetraphosphate (ppGpp) riboswitch also has been reported recently [106]. A T box RNA selectively recognizes a specific non-aminoacylated tRNA as its ligand, which serves as an indicator for a deficit of its cognate amino acid. Uncharged tRNA binding triggers the T box to activate expression of downstream genes that typically encode relevant amino acid biosynthesis genes [107]. A deficit of leucine or other branched-chain amino acids is relevant to conditions that trigger the ‘stringent response’ [108], which leads to the production of the bacterial signalling molecule ppGpp. This signalling molecule has many downstream targets that mediate regulatory pathways to overcome this stress condition, including ppGpp riboswitches [106].

The T box^Leu^ RNA and ppGpp riboswitch domains are observed in either order and utilize separate expression platforms, suggesting that each functions independently of its neighbour. Thus, although T box RNAs are not formally classified as metabolite-binding riboswitches [6,8], the system in either orientation likely functions as a genetic logic gate. Independently, each T box RNA (senses leucine deficiency) and ppGpp riboswitch (senses branched-chain amino acid starvation conditions as well as other nutrient deficiencies) are monitoring for stress conditions that overlap. Furthermore, they can activate similar metabolic pathways when bound to their target ligands. When these two RNAs exist in the same 5ʹ UTR, they likely reduce the ability of either input alone to fully activate gene expression. Instead, the presence of both uncharged tRNA^Leu^ and ppGpp are expected to be needed for full activation of gene expression. If true, then we predict that the system works as a Boolean AND gate [28] (Fig. 2).

Another tandem riboswitch arrangement identified in the present study that forms an AND logic gate involves glycine [27] and c-di-GMP-I [42] riboswitches. Glycine riboswitches in these tandem arrangements are comprised of the typical dual glycine aptamers followed by an expression platform. Although a relationship between these two ligands is uncertain, we speculate that cells carrying this arrangement will commit to a particular physiological change triggered by c-di-GMP only if they have an adequate amount of glycine. Two of the six associated ORFs are relevant to the production and deployment of adhesion proteins curlin [109]. One protein whose gene is associated with the glycine/c-di-GMP tandem system is CsgG, which codes for a lipoprotein involved in the secretion of curlin in *E. coli* [110]. Notably, the *E. coli* curlin protein CsgA is glycine-rich, including multiple runs of four glycine residues, which perhaps creates a high demand for glycine during periods of curlin production.

A link between these two riboswitch ligands is further apparent when considering that the production of curlin is directly controlled by CsgD. This protein is a transcription factor that also regulates the production of AdrA, a diguanylate cyclase enzyme that produces c-di-GMP to signal the biosynthesis of cellulose as another component of the extracellular matrix [111]. CsgD also promotes glycine production by upregulating serine hydroxymethyltransferase activity in *E. coli*, ensuring that glycine pools are sufficient for synthesis of the glycine-rich CsgA [112]. Given these previous findings indicating a strong connection between the levels of glycine and c-di-GMP in some species, it seems likely that tandem glycine/c-di-GMP riboswitches help assess the levels of these molecules to determine if cells should commit to biofilm formation.

#### NOR gates – OFF/OFF

Among the previously reported two-input riboswitch logic gates is a NOR gate [28] configuration formed by tandem SAM-I/AdoCbl riboswitches [25]. Eleven total examples of this tandem were detected in our analysis, including the previously studied example from *Bacillus clausii*. The riboswitches appear in either order, which retains the NOR logic gate function because both riboswitches are predicted to turn off gene expression. This riboswitch system is associated with the *metE* gene that codes for a methyltransferase that converts homocysteine to methionine, which subsequently can be used to make SAM [25]. The NOR gate employs the SAM riboswitch to suppress *metE* expression when SAM is already plentiful. In addition, abundant coenzyme B_12_ also suppresses expression of *metE* via the AdoCbl riboswitch because host cells also carry *metH*, which is a gene coding for an enzyme that uses the coenzyme B_12_ derivative methylcobalamin to more efficiently convert homocysteine to methionine [113,114]. Thus, the *metE* gene is expressed only when SAM levels are low and when coenzyme B_12_ concentrations are inadequate to permit MetH enzyme function.

Another unusual NOR gate arrangement has been reported [115] to involve a SAM-I riboswitch and a preQ_1_-II riboswitch located upstream of a gene annotated as *metK* (SAM synthase). However, we did not rediscover this candidate in our current analysis, and the sequence is no longer evident in genomic databases. Furthermore, additional examples of preQ_1_-II tandem arrangements yielded possible candidates, but these hits failed to appear when conducting the analogous searches with their partner aptamers. Because these candidates might be false positives, they were excluded from the current report.

#### A AND NOT B gates – ON/OFF

Tandem ZTP/THF-I riboswitch arrangements were noted when these riboswitch classes were experimentally validated [89,116]. These tandem systems are predicted to function as A AND NOT B logic gates [28] (Fig. 2), wherein the ZTP riboswitch activates gene expression and the THF-I riboswitch suppresses expression. Examples are found upstream of genes relevant to the biosynthesis of *N*^10^-formyl-tetrahydrofolate (10 f-THF), which supplies the final carbon atom for the closure of the purine ring during the conversion of AICAR (ZMP) into inosine monophosphate (IMP) late in the purine biosynthetic pathway. Thus, when 10 f-THF is deficient, first ZMP and then its triphosphorylated derivative ZTP accumulate [90] to trigger an increase in the expression of genes relevant to 10 f-THF production [89]. Thus, the tandem ZTP/THF-I system signals the cell to produce more folate for the synthesis of 10 f-THF unless its precursor THF is already abundant.

In the current study, we identified five examples of tandem ZTP/glycine riboswitches in several *Bacillus* and *Oceanobacillus* species that had not previously been reported. Together, the tandem ZTP (predicted ON switch) and glycine (predicted OFF switch) riboswitches act as an A AND NOT B logic gate, THF can acquire a formyl group to become 10 F-THF by transfer of the serine side chain to the coenzyme. This yields glycine as a byproduct of the reaction, which provides a metabolic link between the cellular concentrations of ZTP and glycine. Typically, high concentrations of ZTP turn on expression of the adjacent gene [89], which codes for a protein that initiates the conversion of 3-phosphoglycerate into serine for the production of 10 f-THF [117]. However, if glycine is already abundant, expression of this gene will be suppressed even when ZTP levels are high due to the presence of the downstream glycine riboswitch. Glycine can yield single carbon units to THF via the glycine cleavage system.

Six representatives of a tandem Na^+^-I/c-di-AMP riboswitch system were recently uncovered that exhibit A AND NOT B logic function [118]. Specifically, elevated Na^+^ concentrations promote read-through of a transcription terminator associated with the Na^+^ aptamer, whereas elevated concentrations of the signalling molecule c-di-AMP stalls transcription at a second terminator stem. Presumably this logic system allows cells to express genes related to osmotic stress mitigation when Na^+^ concentrations are abnormally high, but only if c-di-AMP concentrations are lowered when cells indeed are experiencing osmotic shock [119,120].

In this study, we identified a total of nine examples of SAM-I riboswitches in tandem with T box^Met^ RNAs that function either as A AND NOT B (two examples) or NOT A AND B (seven examples) logic gates [28], depending on the order of the RNAs within the 5ʹ UTR. The AND and NOR logic gate systems described in the sections above can be formed with riboswitches arranged in either order, which is possible because both riboswitches exhibit the same gene regulation directionality (i.e. ON/ON or OFF/OFF in either scenario). This is not the case for tandem systems comprising one ON and one OFF switch. On ligand binding, the SAM-I riboswitch is predicted to turn off gene expression whereas the T box^Met^ is predicted to turn on gene expression when their respective ligands bind. Because tandems formed from SAM-I and T box^Met^ domains exhibit different directionalities, the order in which the domains appear changes the logic function. Therefore, a T box^Met^/SAM-I tandem creates an A AND NOT B logic gate whereas a SAM-I/T box^Met^ tandem creates a NOT A AND B logic gate as further discussed below. This is the only tandem system we identified that naturally falls under two different logic functions based on the position of each regulatory domain in the 5′ UTR.

#### NOT A AND B gates – OFF/ON

As noted above, two examples of the SAM-I/T box^Met^ were identified that are predicted to function with NOT A AND B logic [28]. Each arrangement precedes genes related to homocysteine and methionine biosynthesis, specifically *metX* and *metY* [113]. Homocysteine can be directly converted to methionine by several different enzymes, and methionine is used as a precursor for SAM biosynthesis. Thus, when SAM concentrations are low and uncharged tRNA^Met^ (an indicator of methionine deficiency) concentrations are high, the system is predicted to permit expression of the genes necessary to produce more methionine and SAM. Conversely, when SAM is abundant or when uncharged tRNA^Met^ is scarce (i.e. most tRNA^Met^ is charged with methionine), expression of these genes is repressed.

A total of 28 examples of tandem TPP/HMP-PP riboswitches [104] were identified that exhibit NOT A AND B logic gate function. HMP-PP (4-amino-5-hydroxymethyl-2-methylpyrimidine diphosphate) is a precursor that is fused to HET-P (5-(2-hydroxyethyl)-4-methylthiazole phosphate) to produce the enzyme cofactor TPP [121]. Tandem TPP/HMP-PP riboswitches are associated with genes relevant for the biosynthesis of HET-P. Thus, the observed logic corresponds well with the need for cells to turn on the production of HET-P if its co-substrate HMP-PP is abundant, but only when the final product TPP is scarce.

We identified five unique examples of adenine riboswitches in tandem with T box^Tyr^ RNAs, all in certain strains of *Clostridium botulinum*. Canonical transcription promoter sequences (−35 and −10 sites) were found upstream of the adenine riboswitch and between the adenine riboswitch and the T box^Tyr^ RNA. When the first promoter is used, both adenine and uncharged tRNA^Tyr^ serve as ligands for a predicted logic gate, whereas only the T box^Tyr^ RNA has regulatory power when the downstream promoter is used. It is currently not known what influences promoter choice.

The adenine/T box^Tyr^ tandem arrangement is located upstream of *tyrZ*, which codes for one of the two tyrosyl-tRNA synthetase proteins in *C. botulinum*. This gene is followed by another coding region for purine nucleoside phosphorylase, which removes the purine base from corresponding nucleosides producing ribose 1-phosphate. It is common for a T box RNA that senses uncharged tRNA^Tyr^ to turn on expression of a gene encoding the enzyme that charges tRNA^Tyr^ with its cognate amino acid. It is also rational for a riboswitch that recognizes adenine to turn off expression of a gene that converts nucleosides into nucleobases as part of the purine salvage pathway. Thus, we predict this system functions as a NOT A and B logic gate. Although the relationship between these two metabolic processes is not obvious, it might be related to the fact that the concentrations of phosphorylated ribose derivatives affect aromatic amino acid biosynthesis [122].

We found two examples of a glycine riboswitch in tandem with a T box^Ser^ RNA. This tandem arrangement is found upstream of genes involved in serine biosynthesis, specifically *serC, serA*, and *serS*. The glycine riboswitch is formed using two full aptamers and likely functions as an OFF switch. The T box^Ser^ senses the depletion of serine by recognizing uncharged tRNA^Ser^ and is predicted to function as an ON switch. Whereas glycine riboswitches typically control glycine biosynthetic and salvage genes [27], some examples have previously been found to control *serC* [123]. Glycine and serine can be interconverted through serine hydroxymethyltransferase [124], and therefore excess glycine can be converted into serine. This provides support for the prediction that the glycine/T box^Ser^ NOT A AND B logic system would activate expression of genes in the serine biosynthetic pathway only if both glycine and serine levels are low.

We identified two examples of AdoCbl riboswitches in tandem with T box^Val^ RNAs that we predict may operate as another example of NOT A AND B logic systems. The distance from the AdoCbl riboswitch to the downstream the T box^Val^ is relatively long, making it more difficult to evaluate this system compared to other tandem arrangements. However, there is no clear open reading frame or additional promoter sequence in the intervening region which leads us to believe that these RNAs are present in the same 5ʹ UTR and both regulate the same open reading frame. In both examples of this proposed AdoCbl/T box^Val^ tandem, found in species in the genus *Blautia*, the resulting mRNA encodes the valyl tRNA synthetase. Although it is common for a T box RNA to upregulate the expression of the synthetase when its cognate tRNA species is non-aminoacylated, it is less clear why levels of AdoCbl (coenzyme B_12_) would be used as a secondary input for this decision. One potential link is that methylmalonyl coA is part of the branched chain amino acid biosynthetic pathway (including valine biosynthesis) and methylmalonyl CoA is also processed by a coenzyme B_12_-dependent enzyme.

Lastly, NOT A AND B logic systems involving AdoCbl riboswitches in tandem with c-di-GMP-I or c-AMP-GMP riboswitches are further discussed in a later section.

#### IMPLY gates – OFF/ON with one shared expression platform

Examples of tandem guanine/PRPP riboswitch aptamers have previously been bioinformatically and experimentally validated as IMPLY Boolean logic gates [56]. Initially, one might think that this arrangement of a guanine riboswitch (OFF) followed by a PRPP riboswitch (ON) should fall into the category of NOT A AND B logic gates, as described above. However, this tandem system is unique in that the guanine and PRPP aptamers are not separated by an intrinsic terminator stem (Fig. 1D). Instead, they appear to affect gene expression by exploiting a single terminator stem that partially overlaps the PRPP aptamer. This specific arrangement of directly adjacent aptamers responding to two different ligands drastically changes the gene expression output profile (Fig. 2) compared to a NOT A AND B logic gate formed by an OFF switch followed by an independent ON switch. Although the precise structural changes exploited by the system to bring about logic gate function have yet to be established, the OFF function typically triggered by guanine binding are overridden by PRPP (phosphoribosyl pyrophosphate) binding to its aptamer [56]. This riboswitch architecture is used to regulate the expression of genes associated with the production of inosine monophosphate and eventually ATP. Under normal circumstances, excess guanine is detected as an indicator to reduce the production of purine-containing nucleotides. However, this signal is countermanded if amino acid starvation causes PRPP (the precursor to the purine biosynthetic pathway) to become elevated, which induces the production of additional ATP [125].

#### An unusual series of logic gates

Within a seven kilobase region of the bacterium *Desulfobacca acetoxidans* reside seven distinct riboswitch representatives from three classes: three c-di-GMP-I riboswitches [42], three AdoCbl riboswitches [1], and one c-AMP-GMP riboswitch [11,12] (Fig. 3A). These RNAs form three clusters separated by one or more ORFs, suggesting that the riboswitches assemble to form three independent logic devices. The aptamers of the AdoCbl riboswitches conform to the reported consensus model [1,6], suggesting that they indeed respond to AdoCbl rather than AqCbl [126]. Furthermore, each AdoCbl aptamer is followed by an intrinsic terminator stem, indicating that the riboswitches perform independently of the adjacent riboswitch.

The c-di-GMP-I aptamers in all three clusters are highly similar, but the aptamer in cluster 3 carries a key nucleotide change within its ligand-binding pocket (Fig. 3B). This nucleotide difference is commonly observed in c-di-GMP-I riboswitch variants that respond to the signalling molecule c-AMP-GMP [10,11], and the nucleotide at this position is known to directly interact with the G or A base of these signalling molecules [43]. Consistent with predictions based on these sequence patterns, the aptamers in clusters 1 and 2 strongly favour c-di-GMP binding (Fig. 3C) as determined by in-line probing assays [40,41]. In contrast, the homologous aptamer in cluster 3 exhibits a marked improvement in c-AMP-GMP binding affinity and perhaps a slight weakening of its affinity for c-di-GMP. Thus, it is possible that the logic gate formed by cluster 3 responds to AdoCbl and c-AMP-GMP.

We predict that the AdoCbl riboswitches are genetic OFF switches and that the c-di-GMP and c-AMP-GMP riboswitches are genetic ON switches. If correct, then these tandem RNAs each function as Boolean NOT A AND B gates. The first cluster also has an additional c-di-GMP-I riboswitch located ~320 nucleotides upstream of the first AdoCbl riboswitch, and its role in the function of the system is currently unclear. Interestingly, c-AMP-GMP is known to regulate bacterial processes involved in exoelectrogenesis [12], which is a process wherein electron-conducting pili are formed. It is known that pili carrying regularly spaced haem prosthetic groups are involved in electron conductance in some species [127]. Perhaps in *Desulfobacca acetoxidans*, there is a link between the formation of conductive pili and the amount of coenzyme B_12_, which carries a corrin ring that is chemically similar to haem.

### Riboswitches and ribozymes

In very rare instances, riboswitch aptamers are located adjacent to ribozymes where they allosterically regulate catalytic function. Note that such arrangements are distinct from that observed for *glmS* ribozymes [128], wherein glucosamine-6-phosphate is bound as a cofactor to promote RNA strand scission rather than serving as an allosteric effector molecule [129,130]. The only natural unimolecular RNA system known to function as a true allosteric ribozyme involves the interplay between c-di-GMP-II riboswitch aptamers and group I ribozymes in the bacterial species *Clostridioides difficile* (formerly *Clostridium difficile*) [59,61]. There are instances of this cooperative system in many *C. difficile* strains, though remarkably we were unable to identify other instances of c-di-GMP-II riboswitches in tandem with group I ribozymes in any other species of bacteria.

The mechanism of this unusual riboswitch arrangement involves the binding of c-di-GMP to a c-di-GMP-II aptamer, which then allows the adjacent group I ribozyme to properly exploit its normal 5′-splice site to yield a processed mRNA that can be translated. However, the ribozyme also requires the binding of guanosine (or any of its 5′-phosphorylated derivatives) to carry out this first step of splicing. Thus, the system operates as a two-input Boolean AND gate wherein both c-di-GMP and guanosine are necessary to express the adjoining ORF [59,61].

To explore the intriguing possibility that additional classes of natural allosteric ribozymes exist in bacteria, we searched for riboswitches and other regulatory RNAs directly upstream of several classes of small self-cleaving ribozymes. Specifically, the genomic contexts of hammerhead [131], HDV [132], twister [32], twister sister [133], pistol [134], and hatchet [135] ribozymes, including their structural permutations, were analysed. Remarkably, not a single example of a riboswitch (or riboswitch aptamer) was found within 2 kilobases of a self-cleaving ribozyme class [136].

If riboswitches do associate with self-cleaving ribozymes, they are certainly very rare and might only occur with yet-to-be-discovered classes of riboswitches and ribozymes. It seems very unlikely that allosteric ribozyme function would have no practical value to modern cells, or that RNA is somehow incapable of forming these devices. Recently, the potential value of allosteric ribozymes was described in detail, along with numerous reasons why they might be vastly underrepresented in extant species [136]. Perhaps most importantly, many of the functions that could be derived by employing an allosteric ribozyme can be attained by using simpler riboswitch architectures.

Searches for tandem riboswitch-ribozyme partnerships can also lead to potential false positives. For example, we uncovered a tandem arrangement between a SAM-I aptamer and a group II self-splicing ribozyme, which could indicate that the SAM-I aptamer uses the ribozyme as an expression platform. However, this possibility seems unlikely because there is an uninterrupted terminator stem following the aptamer. Further analysis revealed that the ribozyme is a member of group IIC ribozymes, which can be components of selfish genetic elements that remarkably are capable of inserting adjacent to intrinsic terminator stems [137,138]. This capability allows them to avoid disrupting ORFs, and this have less of a chance to cause an evolutionary disadvantage to their hosts.

## Riboswitch classes that lack tandem arrangements

Numerous riboswitch classes show no evidence of forming tandem riboswitch arrangements (Table 3). There are several possible reasons for the absence of tandem architectures, and each riboswitch class would need to be considered individually to assess why such configurations are unlikely to occur. For example, we detected a total of 783 representatives of the NiCo riboswitch class [50] without observing any tandem systems. However, the unique structure of the aptamer domain of this riboswitch class forms up to four separate but cooperative binding sites that are selective for Ni^2+^ or Co^2+^ [50], or possibly also for Fe^2+^ [139]. Thus, it seems unnecessary to stack two or more complete NiCo riboswitches to create a pseudo-cooperative system when each representative is already highly cooperative. This might also be the reason why pseudo-cooperative riboswitches for THF-I [44,116] and guanidine-II [70–72] are not observed, as they already cooperatively bind two ligands (Table 1). However, this does not explain the surprising lack of tandem Mn^2+^ riboswitches [140,141], despite the fact that there are over 5,000 representatives of this class [6].

**Table 3.** Riboswitch classes that have not been found in tandem arrangements.

Coenzymes	RNA Derivatives	Elemental Ions	Others
AqCbl	ADP PRA PreQ_1_-I PreQ_1_-II PreQ_1_-III	Mn^2+^ Mg^2+^-II Na^+^-II	Azaaromatic Guanidine-I Guanidine-III Guanidine-IV *glmS*
NAD^+^-II	Xanthine-I Xanthine-II 2’-dG-I	Fluoride NiCo	
SAH	2’-dG-II		
SAM-SAH SAM-III SAM-VI THF-II	2’-dG-III		

Other reasons are possible for the lack of otherwise plausible tandem riboswitch arrangements. For example, given that AdoCbl riboswitches are sometimes naturally used to regulate the expression of genes responsible for Co^2+^ homoeostasis [142,143], a mixed tandem arrangement between NiCo and AdoCbl riboswitches might be expected to exist. The absence of such arrangements could be due several reasons, including the possibility that NiCo riboswitches might preferentially respond to Ni^2+^, Fe^2+^, or Zn^2+^ instead of Co^2+^ [139].

We observe a general trend that more abundant riboswitch classes tend to be found in tandem arrangements. For example, nine out of the ten riboswitch classes with the most natural representatives [6] are found in at least one type of tandem arrangement. The exception is the Mn^2+^ riboswitch class as noted above. Perhaps this is not surprising because more common riboswitches have more opportunities to form pseudo-cooperative arrangements by simple chance. Perhaps for the same reason, the least abundant riboswitch classes tend to lack tandem representatives, including those listed herein (Table 3). However, some uncommon riboswitch classes are found in tandem with another riboswitch class with surprising frequency, such as the HMP-PP [105] and PRPP [56] classes, which could hint at ancient origins for these tandem systems. Regardless, the probability that a riboswitch class will be observed in a tandem arrangement is likely based both on its relative abundance and the biological necessity for gene regulation characteristics that require tandem-arranged riboswitches from this class.

## Concluding remarks

The findings from our bioinformatics searches reinforce the hypothesis that RNA World organisms could have exploited the modular character of RNA structures to create a great diversity of sophisticated molecular sensors and switches [15,144]. Perhaps what we see in modern organisms provides only a modest sampling of the diversity of tandem riboswitch arrangements that would have been used by the earliest forms of life to monitor their environments, regulate their metabolic and physiologic processes, and gain the molecular complexity necessary to give rise to proteins via the evolution of ribosomes. Regardless, it is evident that many bacterial species sense important metabolites and tune their dose-response curves by employing tandem riboswitch systems. In addition, various species also achieve two-input logic decisions by stacking independently functioning riboswitches or by routing the function of two different riboswitch aptamers through a single expression platform.

The opportunities for discovering additional tandem riboswitch arrangements are abundant, given that there exist many ‘orphan’ riboswitch candidates whose ligands have yet to be discovered [35,145–147]. Indeed, we identified many examples where representatives of experimentally validated riboswitch classes are associated with orphan riboswitch candidates. However, we have not discussed these examples in the current report due to the uncertainty regarding the functions of the unvalidated motifs in terms of their ligand binding capabilities as well how that ligand would influence gene expression. In addition, the ever-expanding collection of sequencing data will certainly enable identification of more tandem riboswitch examples. This view is supported by the numerous potential tandem candidates identified in metagenomic and environmental sequencing reads that we excluded due to insufficiencies in either the quality of the sequencing/assembly or genomic context surrounding the potential tandem arrangement.

One key observation regarding the use of riboswitches to form Boolean logic gates is evident from the current natural architectures of tandem riboswitches (Fig. 2). All four possible truth tables that could be created by tandem riboswitch assemblies wherein the riboswitches work independently of each other are naturally represented (AND, NOR, A AND NOT B, NOT A AND B), some with multiple arrangements. Of the six remaining logic gate types that could conceivably be formed by tandem riboswitches, each requires the RNA domains to functionally interact to create the genetic outputs that match the truth tables. Presumably, it is structurally more demanding on the RNAs to achieve this additional functional sophistication, which might in part be why only one of these logic gates (IMPLY) is observed in nature.

The remaining five logic functions that have not been naturally observed (XOR, OR, XNOR, A OR NOT B, and NAND) would probably create favourable gene regulation patterns under certain circumstances. We do not currently have a clear understanding of how XNOR or XOR gates could be constructed using RNA. However, OR gates could result from two ON switches that interact with each other. We also hypothesize that an A OR NOT B gate could be the inverse of the IMPLY logic gate and comprised of an ON followed by OFF interacting aptamers (Fig. 2). NAND are particularly intriguing given that any other logic gate can be created by the coordinated function of multiple NAND gates^28^. Although a NAND gate could result from the interaction of two OFF switches, the architecture needed to achieve this is likely to be more complex than for other logic gate types wherein the tandem riboswitches do not need to interact. Furthermore, we speculate that cells are unlikely to use multiple riboswitch NAND gates to assemble logic devices that can be achieved with simpler assemblies given the additional evolutionary burdens this would create.

The continually expanding collection of natural riboswitches offers opportunities to ‘reverse engineer’ many RNA devices to learn what structures and mechanisms are used to yield high-performance molecular sensors and switches. In general, synthetic biologists have used design strategies that are very different than those optimized through billions of years of evolution. Initial designs for synthetic RNA switches were based on the fusion of aptamers to structurally sensitive parts of the interiors of ribozymes [148–151]. Although our bioinformatics search strategy might miss some examples of these architectures, we have yet to uncover any analogous natural allosteric ribozyme structures [136]. Perhaps synthetic biologists would be better served by mimicking the mechanisms used by natural riboswitches to create novel versions for practical applications. Finally, the missing natural RNA logic gates might be able to be rationally designed using existing riboswitch architectures.

## Data Availability

All data used to generate the conclusions of the study are either cited, presented herein, or available as supplementary materials.
